# Dietary intake, nutritional status and healthcare characteristics of mothers and newborn infants in a prospective cohort study (CHAMP) from a malnutrition-endemic region of Pakistan

**DOI:** 10.3389/fnut.2026.1785862

**Published:** 2026-04-13

**Authors:** Muhammad Shahzad, Ahsan Saidal, Muhammad Ismail, Kalsoom Tariq, Alaa’ Lutfi Melhem, Khalid Iqbal, Maria Ishaq Khattak, Habab Ali Ahmad, Muhammad Saeed, Mustajab Ghani, Ziad Al Nabhani, Simon C. Andrews

**Affiliations:** 1 Faculty of Dentistry, Zarqa University, Zarqa, Jordan; 2 Institute of Basic Medical Sciences, Khyber Medical University, Peshawar, Pakistan; 3 KMU Institute of Health Sciences, Swat, Pakistan; 4 Department of Biochemistry, Khyber Girls Medical College Peshawar, Peshawar, Pakistan; 5 Epidemiological Methods and Etiological Research, Leibniz Institute for Prevention Research and Epidemiology, Bremen, Germany; 6 Institute of Public Health and Social Sciences, Khyber Medical University, Peshawar, Pakistan; 7 Department of Biological and Health Sciences, Pak-Austria Fachhochschule Institute of Applied Sciences and Technology, Haripur, Pakistan; 8 Department of Visceral Surgery and Medicine, Bern University Hospital, Bern, Switzerland; 9 Maurice Müller Laboratories, Department for Biomedical Research, University of Bern, Bern, Switzerland; 10 School of Biological Sciences, Health and Life Sciences Building, University of Reading, Reading, United Kingdom

**Keywords:** birth cohort, breastfeeding practices, dietary diversity, gut microbiome, infant growth, malnutrition, maternal nutrition, rural Pakistan

## Abstract

**Background:**

Dietary intake, nutritional status, healthcare access, and early-life exposures play a critical role in shaping infant growth and development. Recent evidence suggests that the impact is largely mediated by gut microbiome. The Child Health, Nutrition and Microbiome in Pakistan (the CHAMP study) is a longitudinal cohort study aiming to assess the bidirectional relationship between gut microbiome and infant growth and development in a cohort of children residing in malnutrition endemic areas of Pakistan.

**Objectives:**

The current study report the baseline sociodemographic, dietary, healthcare, and nutritional characteristics of mother–infant dyads participating in the CHAMP study.

**Methods:**

Baseline data were collected from 70 mothers and 72 newborn infants recruited from rural communities in District Swat, Pakistan. Information on household socioeconomic status, maternal dietary intake, antenatal, delivery and postnatal care, infant feeding practices, morbidity, and anthropometric measurements was obtained using validated tools. Descriptive and sex-stratified analyses were conducted.

**Results:**

Households were socioeconomically disadvantaged, with low parental education, large family size, and mean household income substantially below the national minimum wage. Maternal utilization and quality of antenatal and postnatal care were suboptimal, including limited completion of recommended antenatal visits. Dietary quality was also poor, and none of the mothers met the recommended minimum dietary diversity for women. Among infants, morbidity was common, with nearly half experiencing diarrheal illness or respiratory infections. Exclusive breastfeeding reported only in 43.1% of infants. Anthropometric assessment revealed evidence of early growth faltering, with 35.4% of infants were low-length-for-age with higher prevalence among females.

**Conclusion:**

These baseline findings highlight substantial socioeconomic vulnerability, poor maternal diet quality, gaps in maternal and infant healthcare, and early-life undernutrition in this rural Pakistani cohort. The study finding provides foundation for longitudinal analyses examining how these factors interact with gut microbiome development and child growth, informing and cost effective and culturally relevant intervention strategies.

## 1 Introduction

Maternal dietary intake and nutritional status during pregnancy and the early postnatal period play crucial roles in shaping birth outcomes and child health trajectories, as reported in “The Lancet” land mark series on maternal and child nutrition (1, 2). There is thus systematic evidence of the importance of adequate nutrition during the first 1,000 days of life, from conception to the second birthday. During this time period also known as “the window of opportunity” (3), adequate nutrition is critically important for physical and mental growth, overall well-being and a healthy life (4, 5). Poor nutritional status or malnutrition during this period is a major determinant of impaired growth, increased morbidity and long-term developmental deficit in children (6). This issue is particularly prevalent in Low and Middle-income Countries (LMICs), where nearly half of all deaths in children under 5 years of age is due to malnutrition (7).

Pakistan is among those countries suffering from a consistently high burden of maternal and child malnutrition over the last several decades. The country rank among the top five countries in the world with high prevalence of stunting, wasting and micronutrient deficiencies in children under 5 years of age (8). Within the country, rural and remote areas are particularly at high risk where maternal and child malnutrition, compounded by poverty, food insecurity and limited access to healthcare, further exacerbates the problem. Despite continuous efforts from government and developmental organizations over recent decades, Pakistan has shown very little or no progress in improving the nutritional status of the population (8).

Recently, increasing attention has been directed toward the role of the gut microbiome in child health, growth and development. Emerging research evidence suggests that maternal dietary intake, nutrition and health status during pregnancy and the postnatal period help in shaping the gut microbiome of infants which in turn modulates immune development, growth and lifelong development (9). Adeptly addressing childhood malnutrition therefore requires nutritional intervention strategies that consider the gut microbiome in order to achieve optimal and sustainable health effects (10). However, designing and implementing effective microbiome directed nutritional strategies requires contextualization of microbiome data with underlying social, environmental, dietary and healthcare characteristics of both the mother and infants during crucial developmental periods. In Pakistan, despite the high burden of malnutrition, available data on maternal and child health, diet, nutrition and healthcare during pregnancy and the postnatal period are scarce and are predominantly derived from cross-sectional surveys or healthcare-program planning and evaluation. In this context, prospective birth cohort studies provide powerful alternatives for generating high quality data within a well-defined social or geographical context such as rural and remote areas with high prevalence of malnutrition.

The present study is embedded in our ongoing longitudinal study on a birth cohort (the CHAMP study) of infants from rural Pakistan where childhood malnutrition is prevalent and multifactorial in origin (11). The overall aim of the study is to longitudinally characterize gut microbiome development, and its association with growth and development of the newborn infants during the first 2 years of life. Here, we report the cohort profile and the baseline characteristics of participating mothers and infants, alongside environmental and household factors, prior to longitudinal follow-up. These factors are hypothesized to affect gut microbiome development and maturation during early infancy and childhood. Although, the present study only reports the baseline characteristics of the CHAMP cohort, establishing this baseline is a critical step for assessing the upstream determinants that will be analyzed in relation to subsequent longitudinal microbiome analyses and interpretation in future.

## 2 Methodology

### 2.1 Cohort description

Detailed information about the CHAMP study design, recruitment strategy, data and sample collection methods have been published elsewhere (11). The following sections briefly summarize the cohort profile and provide updates on the current status of the study.

### 2.2 Study design, setting and participant recruitment

The CHAMP cohort study is being conducted in remote, rural communities of District Swat, located in Khyber Pakhtunkhwa province of Pakistan. The study participants were recruited from three randomly selected village councils (primary sampling units) across the study site from May to June 2024. A total of 93 mothers who have given birth to a child during the last 28 days were invited to participate in the study with the help of Lady Health Workers. Of these, 23 dropped out prior to baseline data collection due to various reasons, the commonest being no permission granted from male head of the household. The remaining 70 women were included in the study and thus data and biospecimens samples were collected from these participants during the first study visit (baseline). Mother-infant dyads will continue data over the following 2 years. Ethical approval for the study was obtained from the Ethics Board of Khyber Medical University, Pakistan (Ref no: DIR/KMU-EB/BR/001-03). The study is also registered on clinicaltrials.gov via Ref no: NCT05793294.

### 2.3 Data collection

Household, maternal and infant related data were collected by trained research assistants using structured, validated questionnaires. Household data were collected from mothers and include demographic characteristics and socioeconomic profiles (family size, presence of kitchen and toilet, number of bedrooms, educational attainment and profession of household head, monthly household income, assets and amenities, and drinking water sources). Maternal baseline characteristics include age, education level, ante-natal care, delivery and postnatal care characteristics, dietary intake and physical health (height, weight and BMI). The infant baseline data include age (in days), gender, mode of delivery and immunization record. At baseline, mothers were also asked to report infant health-related events including respiratory tract infections (RTI), diarrheal episodes and antibiotics use since birth. RTIs were defined as episodes of cough and/or difficulty in breathing, with or without fever (12) while diarrhea was defined as passage of ≥3 watery stools in 24 h period (13). Information about antibiotic exposure of infants were also collected from mothers using structured questionnaire. Mothers were also asked to provide information about infant feeding practices using validated, infant and young child feeding practices (IYCF) questionnaire developed by WHO (14).

### 2.4 Anthropometric measurements and data quality

Anthropometric assessments on both the mother and the infants were performed during household visits up to 28 days following childbirth. Briefly, the infant heigh was measured by requesting the mother to gently put the baby on an infantometer such that the infant head is aligned against the headboard. A research assistant then straightens the infant body and feet, move the footboard firmly against the child heels and record the length to the nearest 0.1 cm. Maternal height was measured by instructing the mother to remove shoes and standing steadily on the height stadiometer in Frankfurt plane position. Once ready, the research assistant gently pushes the moving headboard down until it touches the crown of the person’s head. The height is recorded to the nearest 0.1 cm. To record infant weight, the mothers were instructed to remove shoes and heavy clothing from the baby and place him/her on the center of the specialized electronic pan scale (Secca, UK). Weight was recorded to the nearest 0.1 kg when the infant was still in the pan and digital display does not change anymore. Mother weight was measured in kg using a calibrated electronic weight machine after removing shoes, jewelry and extra clothing (dupatta/chaddar). The anthropometric measurements of the infant were entered into WHO Anthro version 3.2.2 to calculate height-for-age z-score (HAZ) and weight-for-age z-score (WAZ). Low length-for-age and weight-for-age was defined as HAZ and WAZ < −2 SD from the median value of WHO reference data. To assess maternal nutritional status, body mass index (BMI) was computed as the fraction of weight to the squared height (kg/m2) and classified as Underweight (BMI = <18.5), normal (BMI = 18.5–22.9), overweight (BMI = 23–27.5) and obese (BMI = ≥ 27.5) based on WHO cut off for BMI in Asian women. For assessment of data quality, the raw anthropometric data (both hard and soft files) were double-checked and verified for errors. Biologically implausible values were also identified according to WHO growth standards. To assess the impact of extreme values on anthropometric growth assessment, we have conducted sensitivity analysis by excluding the values that were clearly incorrect or biologically implausible.

### 2.5 Biospecimens collection

At baseline, dry blood samples (DBS) were collected from both mother and infants by skin puncture of the third or fourth finger following standard procedures (15). DBS were transported to the main lab and stored until further processing and analysis. Due to cultural barriers, stool samples were only collected from the infants. For this purpose, mothers were instructed to transfer fresh stool samples from baby diapers into sample collection pots which were placed into zip lock bags and then handed over to the research assistant within 4 h of collection. The samples were aliquoted, mixed with DNA preservative (DNA Shield, Zymo Research United States) and stored at −80 °C till further processing.

### 2.6 Statistical analysis

Categorical variables were summarized as frequencies and percentages, and continuous variables as means ± standard deviations or medians with interquartile ranges, as appropriate. Group comparisons used Fisher’s exact tests for categorical data and Mann–Whitney U tests for continuous data. Analyses were conducted using SPSS version 27 and RStudio (version 2025.05.1 + 513).

## 3 Results

### 3.1 Participant characteristics

Baseline data were collected in May to June 2024 from 72 children (39 males and 33 females) and the corresponding 70 mothers (note that one of the mothers gives birth to triplets and they were included in the study). Table 1 presents the sociodemographic characteristics of the mother–infant dyads stratified by child gender. The mean age of mothers was 27.9 ± 6.6 years, with no significant differences between mothers of male and female child. Parental education level was generally low with 67.1% of mothers and 51.4% of fathers having received no formal education. More than two thirds (*n* = 48; 68.6%) of the households received drinking water supply from natural springs. A significant proportion of the fathers worked as unskilled laborers (*n* = 43; 61.4%). The majority of household belonged to a lower socioeconomic background as evident from the average monthly household income of 18,857 ± 12,084 PKR, which is far below the national minimum wage (35,000 PKR).

**Table 1:** Demographic and socioeconomic characteristics of the study participants at baseline (*n* = 70).

Characteristic	Mothers (*n* = 70)	Male babies (*n* = 39)	Female babies (*n* = 33)	*P*-value
Mother’s age				
Mean (±SD)	27.9 (±6.6)	27.86 (±6.56)	27.97 (±6.74)	0.95
<30 years, *n* (%)	36 (51.4%)	20 (54.1%)	16 (48.5%)	0.81
≥30 years, *n* (%)	34 (48.6%)	17 (45.9%)	17 (51.5%)	
Family size				
Mean (±SD)	6.11 (±2.34)	6.19 (±2.57)	6.03 (±2.10)	0.78
Median [Min, Max]	6 [3, 14]	6 [3, 14]	6 [3, 10]	
1–4 members, *n* (%)	19 (27.1%)	11 (29.7%)	8 (24.2%)	0.82
5–9 members, *n* (%)	44 (62.9%)	22 (59.5%)	22 (66.7%)	
≥10 members, *n* (%)	7 (10%)	4 (10.8%)	3 (9.1%)	
Mother’s education				
No formal education, *n* (%)	47 (67.1%)	19 (51.4%)	28 (84.8%)	<0.001
Primary level, *n* (%)	9 (12.9%)	5 (13.5%)	4 (21.1%)	
High school, *n* (%)	12 (17.1%)	12 (32.4%)	–	
College/University level, *n* (%)	2 (2.9%)	1 (2.7%)	1 (3%)	
Father’s education				
No formal education, *n* (%)	36 (51.4%)	16 (43.2%)	20 (60.6%)	0.31
Primary level, *n* (%)	2 (2.9%)	1 (2.7%)	1 (3.0%)	
High school, *n* (%)	23 (32.9%)	13 (35.1%)	10 (30.3%)	
College/University level, *n* (%)	9 (12.9%)	7 (18.9%)	2 (6.1%)	
Father/Guardian profession				
Agriculture	8 (11.4%)	5 (13.5%)	3 (9.1%)	0.38
Skilled labor	19 (27.1%)	20 (54.1%)	23 (69.7%)	
Non-skilled labor	43 (61.4%)	12 (32.4%)	7 (21.2%)	
Monthly household income (in Pakistani Rupees – PKR)				
Mean (±SD)	18,857 (±12,084)	21,865 (±13,489)	15,485 (±9,540)	0.03
Median [Min, Max]	15,000 [4,000, 50,000]	20,000 [6,000, 50,000]	10,000 [4,000, 45,000]	
<20,000 PKR, *n* (%)	40 (57.1%)	17 (45.9%)	23 (69.7%)	0.06
≥20,000 PKR, *n* (%)	30 (42.9%)	20 (54.1%)	10 (30.3%)	
Household income quartile				
Q1	20 (27.8%)	12 (37.5%)	8 (20.0%)	0.15
Q2	17 (23.6%)	7 (21.9%)	10 (25.0%)	
Q3	18 (25.0%)	9 (28.1%)	9 (22.5%)	
Q4	17 (23.6%)	4 (12.5%)	13 (32.5%)	
Source of drinking water				
Spring water, *n* (%)	48 (68.6%)	23 (62.2%)	25 (75.8%)	0.53
Covered well, *n* (%)	12 (17.1%)	8 (21.6%)	4 (12.1%)	
Municipal/piped water, *n* (%)	5 (7.1%)	3 (8.1%)	2 (6.1%)	
Hand pump, *n* (%)	4 (5.7%)	3 (8.1%)	1 (3.0%)	
Other, *n* (%)	1 (1.4%)	–	1 (3.0%)	

**Figure 1:**
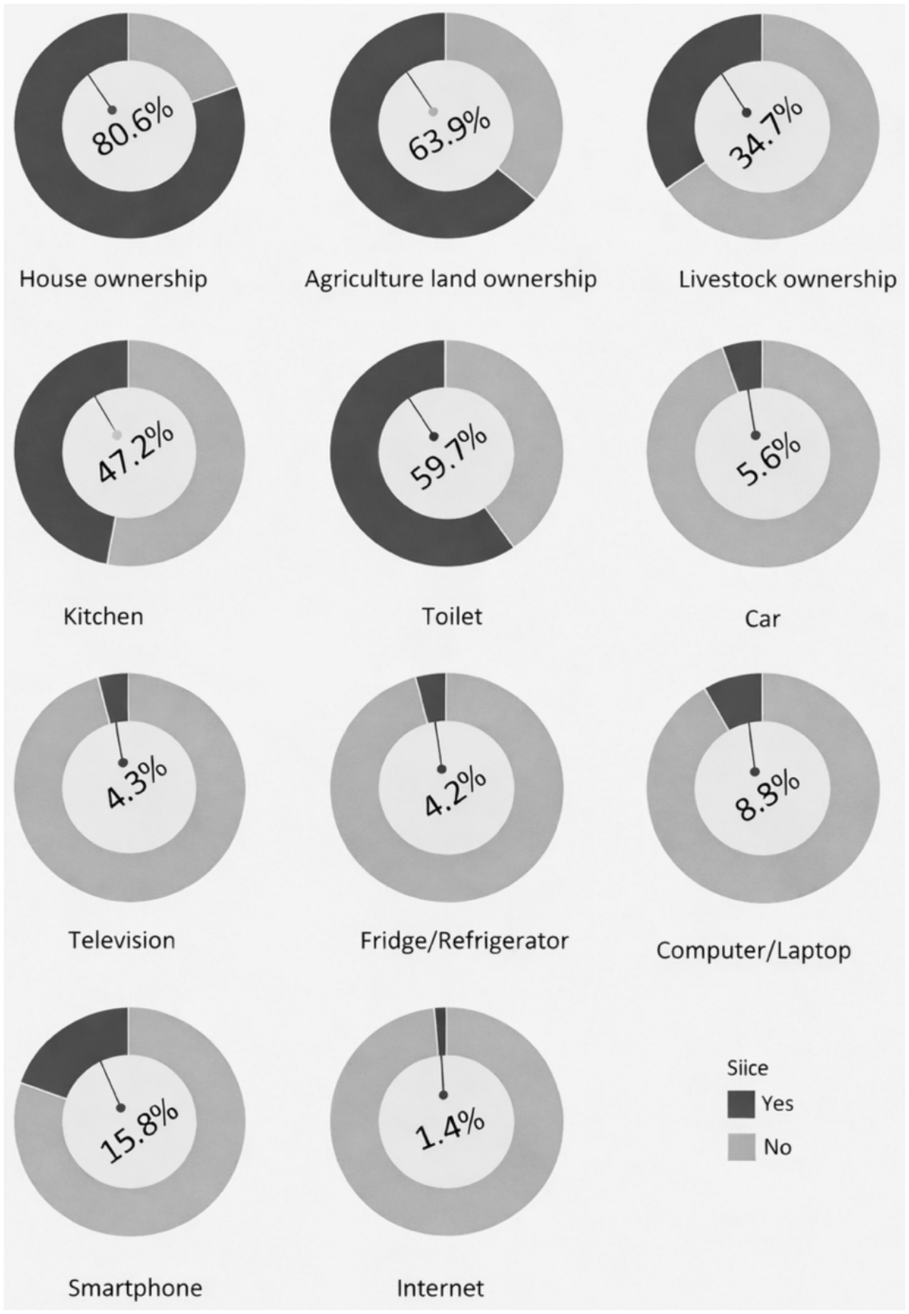
Household amenities and assets.

The socioeconomic status was further confirmed by collecting data regarding household amenities, assets and ownership. As shown in Figure 1, more than 90% of the households do not own any vehicle, television, refrigerator or computer, and have no internet supply indicating limited access to productive resources and modern amenities thus confirming their socioeconomic vulnerability.

### 3.2 Maternal utilization of antenatal and postnatal care services

**Figure 2:**
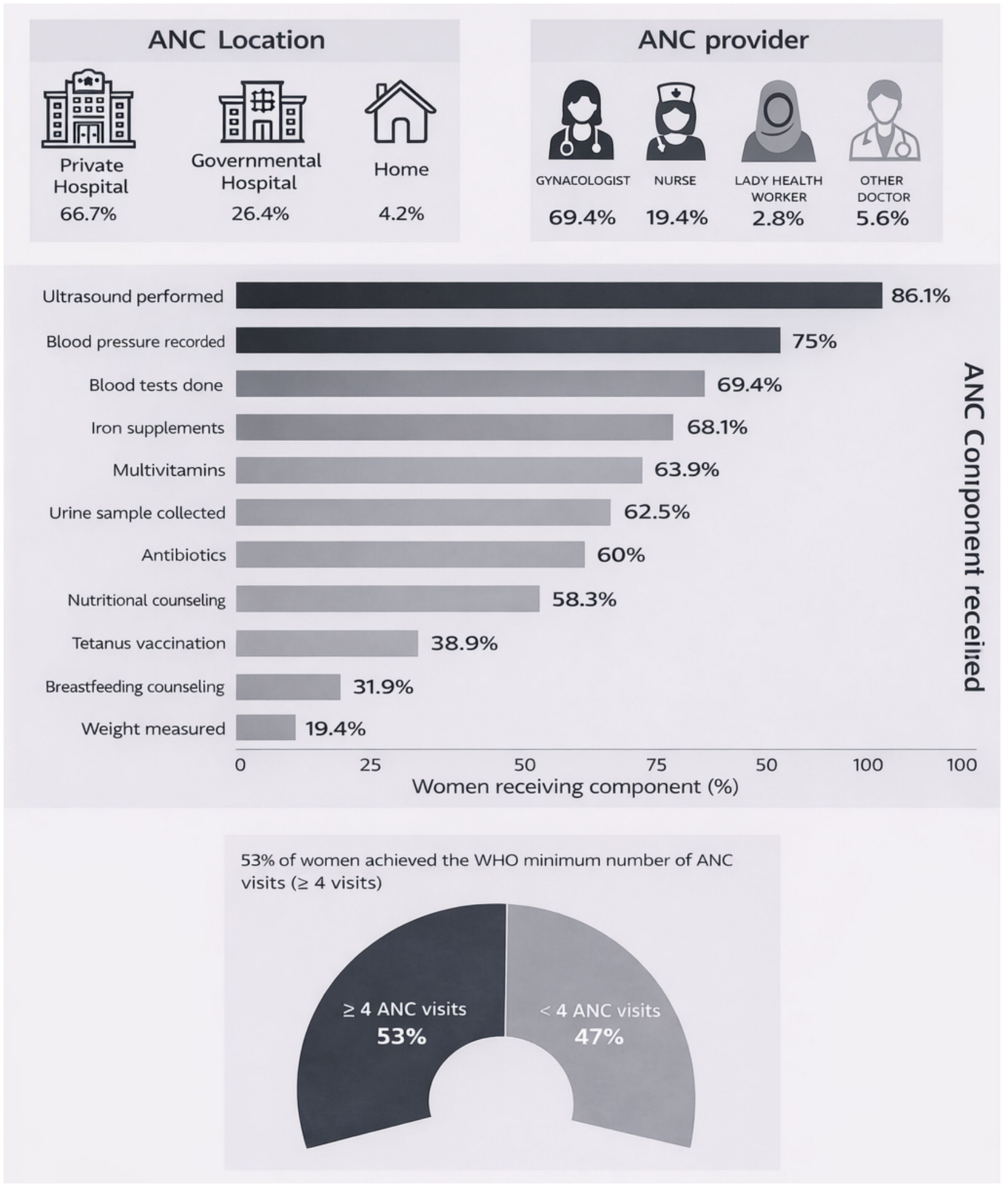
Antenatal care characteristics of the mothers (*n* = 70).

Access, quality and utilization of maternal healthcare services are crucial for the health and well-being of both the mother and her newborn child. Figure 2 presents an infographic summary of the antenatal care characteristics of all mothers (*n* = 70) participating the in the study. Around 70% of the health checks during pregnancy (antenatal care, ANC) were conducted in private hospitals (Figure 2). Other less common healthcare providers for ANC included nurses (19.4%) and lady health workers (2.8%). Of the total ANC visits, around two thirds (66.7%) took place in private hospitals and only 26.4% in government hospitals. Adherence to the recommended minimum four ANC visit was low with only 53% of women having attended four or more ANC visits. Wide variations were reported for physical examination and clinical tests (e.g., weight record, BP measurement, collection of urine and blood samples) during ANC visits. Overall, an ultrasound scan was most frequently conducted test during ANC visits (86.1%) followed by BP measurements (75%). However, the prevalence of receiving proper counseling for nutrition and the importance of breastfeeding was relatively low, and was only reported by 58.3 and 31.9% and of the participating mothers, respectively. Antibiotic use was also common and 43 (61.4%) of the study participants reported using antibiotics at least once during the last pregnancy.

**Figure 3:**
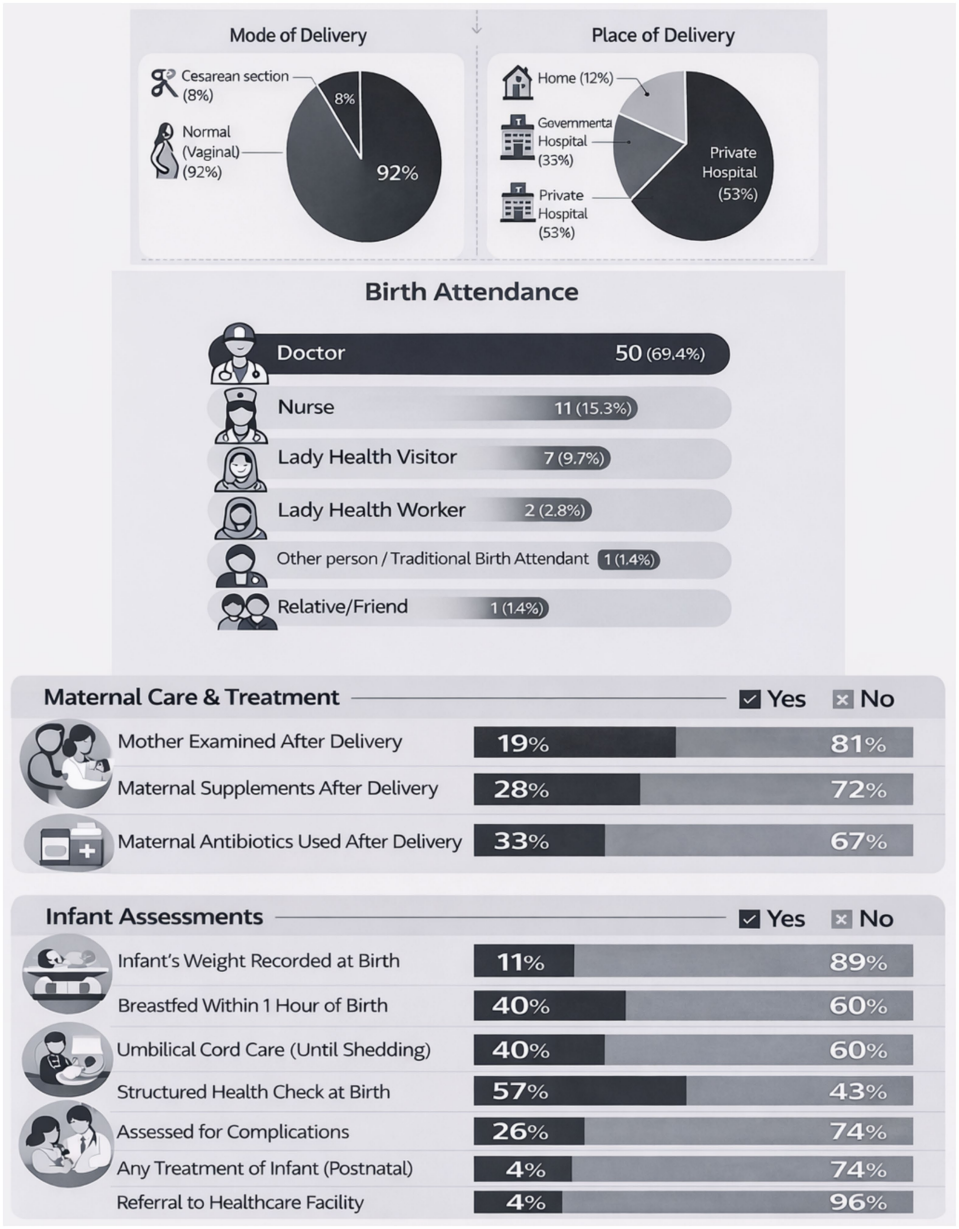
Delivery and postnatal care characteristics of the mothers.

The delivery and postnatal care data of the mothers is summarized in Figure 3. The majority (92%) of babies were born via normal vaginal delivery, with the remainder (just 8%) delivered by cesarean section (C-section). Around half of the deliveries occurred in private hospitals under the supervision of a qualified doctor. At birth, structural health checks took place in around 60% of the babies. Early initiation of breastfeeding within 1 h of delivery was reported for 40% of newborns infants.

### 3.3 Maternal dietary intake and nutritional status

**Figure 4:**
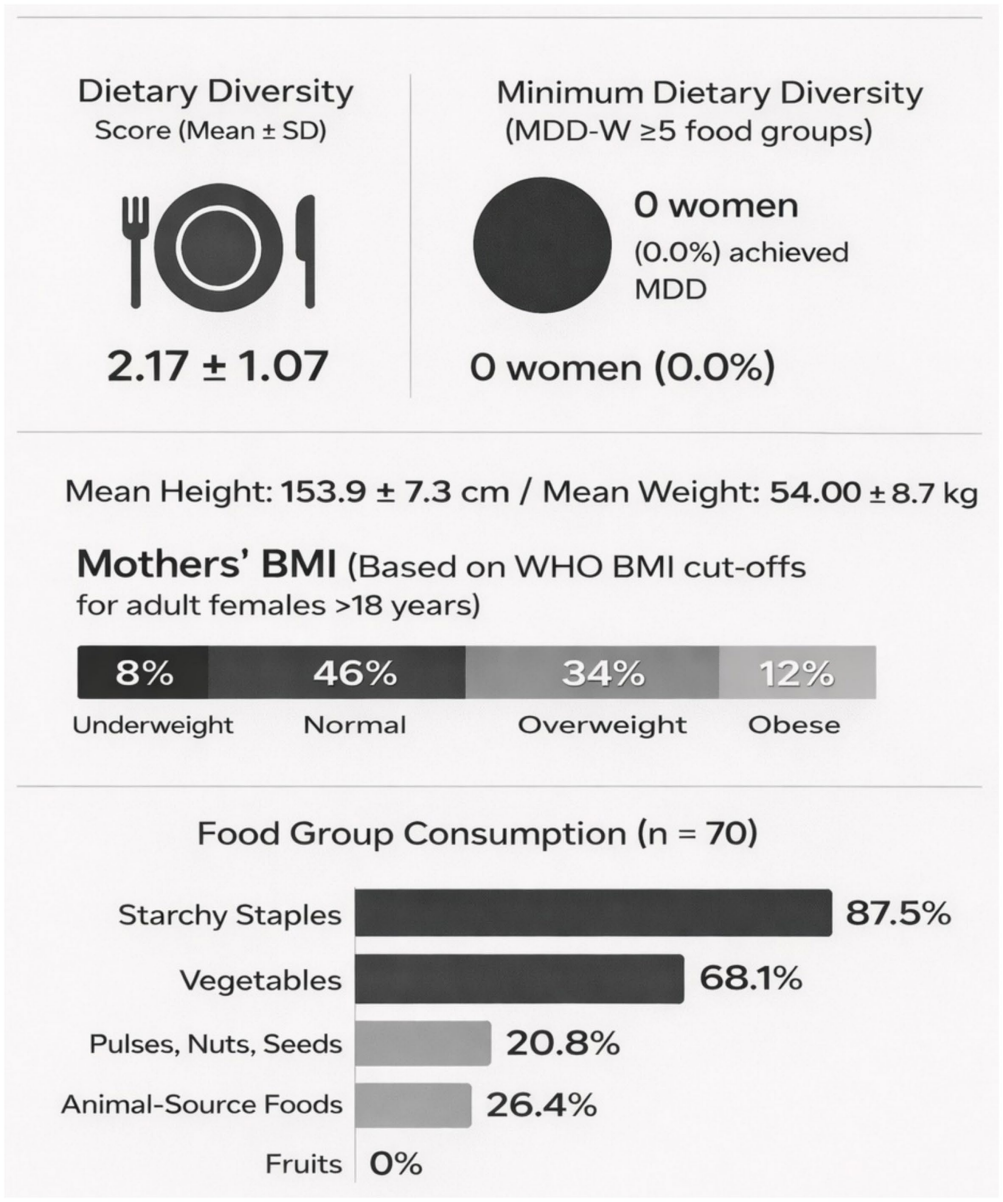
Dietary intake of the mothers as assessed by dietary quality questionnaire (DQQ). DDS = Dietary Diversity Score (range: 1–10). MDD-W = Minimum Dietary Diversity for Women (≥5 food groups in previous 24 h).

Maternal dietary intake assessed using a 24-h recall at the time of data collection showed an overall low dietary diversity score (2.17 ± 1.07) (Figure 4) indicating poor diet quality during the immediate postpartum period. None of the women meet the recommended minimum dietary diversity (MDDW) score of ≥5 food groups during the last 24 h. In terms of food groups, starchy staples were the main foods consumed (87.5%) followed by vegetables (68.1%) and animal source foods (26.4%). The mean height and weight of the mothers were 54.0 ± 8.7 kg and 153.9 ± 7.3 cm, respectively. Based on WHO BMI cut offs for adult females (>18 years), 8% of the mothers were underweight, 34% overweight and 12% were obese. However, BMI classification based on WHO cut-offs may not accurately reflect the nutritional status at this stage since anthropometric measurements were conducted <6 weeks postpartum and therefore, should be interpreted with caution.

### 3.4 Infant health, feeding practices and anthropometric data

The distribution of health, feeding practices and anthropometric data at baseline, stratified by infant gender, are presented in Table 2. Immunization records were available for 40.8% of children overall, with a higher proportion among males (50.0%) compared to females (30.3%). However, the difference was not significant (*p* = 0.092). Respiratory tract infections were reported in 41.7% of children, while 40.8% had received antibiotics since birth, with no significant differences between sexes. Nearly half of the children (47.2%) experienced at least one episode of diarrhea with female infants had a higher median number of episodes [4 (IQR: 2–6)] compared to males [3 (IQR: 2–4)], and the difference was statistically significant (*p* = 0.025). Infant and young child feeding (IYCF) practices showed generally low adherence to recommended breastfeeding practices. Early initiation of breastfeeding was reported in only 22.2% of children, while exclusive breastfeeding during the first 2 days of life was reported at 25.0%. Overall, 43.1% of children were exclusively breastfed with no significant gender-based differences.

**Table 2:** Infant morbidity, feeding practices and anthropometric indicators stratified by sex.

Variable	Overall (*n* = 72)	Male (*n* = 39)	Female (*n* = 33)	*P*-value
Child immunization and health record				
Immunization records available	29 (40.8%)	19 (50.0%)	10 (30.3%)	0.092
Respiratory tract infections (RTIs) since birth	30 (41.7%)	14 (35.9%)	16 (48.5%)	0.280
Antibiotics used since birth	29 (40.8%)	17 (44.7%)	12 (36.4%)	0.474
Diarrhea episodes since birth	34 (47.2%)	16 (41.0%)	18 (54.5%)	0.252
Number of diarrhea episodes since birth [Median, (IQR)]	3.5 (3–6)	3 (2–4)	4 (2–6)	**0.025**

Variable	Overall (*n* = 72)	Male (*n* = 39)	Female (*n* = 33)	*P*-value
IYCF data				
Early initiation of breastfeeding	16 (22.2%)	8 (20.5%)	8 (24.2%)	0.704
Exclusive breastfeeding for first 2 days	18 (25.0%)	11 (28.2%)	7 (21.2%)	0.495
Exclusive breastfeeding	31 (43.1%)	18 (46.2%)	13 (39.4%)	0.564
Mixed milk feeding	18 (25.0%)	10 (25.6%)	8 (24.2%)	0.891
Bottle feeding	20 (27.8%)	11 (28.2%)	9 (27.3%)	0.930

Variable	Overall (*n* = 65)	Male (*n* = 34)	Female (*n* = 31)	*P*-value
Infant anthropometric assessment				
Length in cm (Mean ± SD)	49.27 ± 4.42	50.57 ± 4.51	47.85 ± 3.89	0.27
Weight in Kg (Mean ± SD)	3.66 ± 0.80	3.83 ± 0.73	3.46 ± 0.84	**0.05**
Weight for age z-score	−0.10 ± 1.30	−0.04 ± 1.24	−0.16 ± 1.39	0.52
Height for age z-score	−1.26 ± 2.08	−0.95 ± 2.20	−1.61 ± 1.92	0.17
Head circumference	35.63 ± 4.02	35.58 ± 4.98	35.69 ± 2.54	0.91
Low-weight-for-age	5 (7.7%)	4 (11.8% of male)	1 (3.2% of female)	0.68
Low-length-for-age	23 (35.4%)	10 (29.4% of male)	13 (41.9% of female)	0.31

Bold values indicate statistically significant *p*-values.

Anthropometric assessment indicated a mean length of 49.2 ± 4.4 cm and HAZ score of −1.2 ± 2.08 with no significant difference between boys and girls at baseline. Initial data analysis displayed wide dispersion in length and HAZ score (standard deviation of 7.7 and 2.36 respectively). However, following data quality assessment and removing incorrect and biologically implausible values (*n* = 7), variability in length decreases substantially (SD = 4.4). However, the standard deviation of HAZ remained relatively high (SD = 2.06) suggesting additional factors contributing to the variations HAZ score. The mean weight of infants was 3.66 ± 0.80 kg at baseline. Male children had a higher mean weight compared to females (3.83 ± 0.73 kg vs. 3.46 ± 0.84 kg), with this difference approaching statistical significance (*p* = 0.05). Mean WAZ and HAZ scores were below zero for the overall sample, suggesting a general trend toward suboptimal growth, although no significant sex-based differences were observed. The prevalence of low-weight-for-age was 7.7%, while low-length-for-age was observed in one-third of the children (35.4%), with a higher proportion among females (41.9% %) than males (29.4%).

## 4 Discussion

This current study presents the baseline characteristics of the CHAMP cohort study, which recruited 72 neonates (39 males and 33 females) and 70 mothers from a rural and low resource community in Pakistan. The results underscore significant differences in household socioeconomic status and parental attributes, deficiencies in antenatal, delivery, and postnatal care, as well as notable obstacles in maternal and infant diet and nutritional status.

Overall, households exhibit large family size, poor socioeconomic status and low parental education. Education attainment of the mothers was particularly low with two third of the mothers have no formal education. These finding align with evidence from developing countries with significant implications for the child health and development (16). For example, there is considerable research evidence confirming that higher maternal education during the first 1,000 days of life is strongly associated with improved child survival, growth, and healthcare utilization (17). The same trend is also true for Pakistan, where research consistently shows that maternal illiteracy is linked to poorer child health outcomes, especially in rural areas (18–20). The educational disadvantage observed in mothers not only reflect economic inequality but also entrenched intergenerational inequities, embedded within patriarchal systems of resource distribution in Pakistan (21).

An important determinant of both mother and newborn health is the access, quality and utilization of maternal healthcare services available during the crucial 1,000 days of life. Our study found that approximately half of the completed the minimum four ANC visits recommended by WHO. Essential clinical assessments including blood and urine tests were infrequently performed while and nutritional and breastfeeding counseling was extremely low. These findings align with global evidence from other LMICs (e.g., Uganda, Kenya, Myanmar, Zimbabwe, Somaliland) where inequities in antenatal care coverage and quality remain major barriers to reducing maternal and neonatal mortality (22). Nonetheless, since the World Health Organization (WHO) introduced the ANC model in 2002, recommending a minimum of four contacts during pregnancy, global utilization has gradually improved to 64% between 2007 and 2014 (23). Completion of the recommended visits is strongly influenced by determinants such as maternal literacy, socio-economic status, employment, autonomy, place of residence, and access to health information (24). Similarly in Pakistan, despite improvements in ANC utilization, the quality and consistency of care remain highly variable. For examples, one study reported stark disparities in the recommended four ANC visits, ranging from as low as 12% in Baluchistan and 24% in Khyber Pakhtunkhwa to as high as 82% in Islamabad (25).

Our study also revealed similar gaps in delivery and postnatal care. Overall, over 90% of births occurring by normal vaginal delivery, cesarean sections being rare, and more than half taking place in basic health units or small clinics, while 12% occurred at home. The findings highlight substantial barriers to emergency obstetric care in rural Pakistan, where cesarean section use remains markedly below international benchmarks. In Khyber Pakhtunkhwa, for instance, only 2.1% of births in 1990–91 and 10% in 2017–18 were delivered by cesarean section contrary to 15 and 19% in developed nations, underscoring serious underutilization of this life-saving intervention (26). Early initiation of breastfeeding was also suboptimal, with only 40% of the infants breastfed within the first hour of birth. These findings mirror wider trends reported across South Asia, where research has shown that traditional practices and inadequate counseling within health facilities often delay the first feed. Despite strong evidence of its role in preventing neonatal deaths, initiation rates remain low in the region, averaging around 41 percent and dropping to only 29 percent in Pakistan (27). Although in our study most infants were examined after birth, only 57% received structured health checks, with poor vaccination coverage and inconsistent record-keeping. While postnatal care is central to improving neonatal survival, a cross-sectional study across 23 LMICs found that quality coverage remains limited, reaching only 41% for women and 42% for newborns. Pakistan performed worse than most countries, with maternal and neonatal coverage estimated at 30–35% (28). These findings underscore wider systemic shortcomings, including inadequate resources and gaps within programs such as the Lady Health Worker initiative, highlighting the need for reforms in workforce training, referral pathways, and overall health system integration (29).

Maternal dietary intake and nutritional status was also suboptimal. None of the mother meet the minimum dietary diversity of consuming ≥5 groups of foods with the diet mainly consist of starch staples and vegetables. Due to poor socioeconomic status and literacy rate, these findings are not uncommon and reported in other areas of Pakistan too (8, 30). Poor dietary intake and diversity was also reflected in the nutritional status of the mothers with high prevalence of underweight, overweight and obesity. However, dietary diversity was assessed using a single 24-h recall and represents short-term dietary intake, the finding cannot constitute solid evidence of habitual diet quality or chronic malnutrition.

Our study finding on infant health, feeding practices and nutritional status are also concerning. Morbidity was widespread, with nearly half of infants experienced at least one episode of diarrhea since birth. Diarrheal diseases is the third leading cause of childhood mortality across the world (31) and Pakistan is no exception. Every year, around 60% of the infant and young children deaths in Pakistan occurs due to diarrheal diseases (32). The increase prevalence of diarrhea and associated mortality in all developing countries including Pakistan is generally attributed to unsafe drinking water. In this study, more than two-thirds of households obtained their drinking water from natural springs. These sources are largely unprotected and therefore highly vulnerable to environmental contamination by toxic substances and infectious agents (33, 34). Such exposure may contribute to the high prevalence of diarrheal diseases observed in this population. However, the role of other important factors such as breastfeeding cannot be overruled. IYCF data in this study revealed suboptimal adherence to the recommended feeding practices with only 43.1% of children are exclusively breastfed. This percentage is significantly less than the national average (48%) and WHO recommended target (60%) (35). Low prevalence of exclusive breastfeeding can be due several reasons including but not limited to poverty, lack of education and awareness, reduce counseling during ANC visits and cultural barriers (35).

Anthropometric assessment of the infant revealed gender-based differences with female infant having significantly lower weight compared to male infants. These findings align with evidence from other local evidence in Pakistan showing that son preference and gender bias drives unequal allocation of resources within households (36). Similarly, another larger study across 66 low- and middle-income countries demonstrated that daughters had lower height-for-age (−0.135 SD) and weight-for-age (−0.098 SD) scores compared with sons, with the most pronounced effects among disadvantaged families (37). The gender-based differences were also reflected in the prevalence of stunting with high prevalence among females (40.6%) than males (27.0%). Malnutrition, both stunting and underweight, might also be the reason behind high burden of infections observed in our study as explained by evidence from reviews highlighting the global crisis of malnutrition, which contributes to nearly half of under-five deaths each year (38). This reflects the bidirectional relationship between undernutrition and infectious disease, whereby malnutrition increases susceptibility to illness, while recurrent infections further exacerbate growth faltering.

Our study has several limitations that need to be considered when interpreting the results. First, the anthropometric data of infants in the current study showed substantial variability in height/length and (SD 7.7) HAZ scores (SD 2.36). After excluding the clearly erroneous and biologically implausible anthropometric measurements, the variability in HAZ score remain elevated (SD 2.08) indicating additional factors also play a role. These include difficulties in the precise anthropometric measurements in field conditions, age differences in early life and mixed ages (0–28 days). These findings are indicative of general population pattern rather than precise growth disturbances and therefore, should be interpreted with caution. Second, unavailability of gestational age data further limits our understanding of length-for-age z-scores in infants. As a result, we are not sure whether the whether the low length-for-age z-scores (35%) in our study is due to intrauterine growth restrictions, premature birth or postnatal physiological changes. Third, information about dietary intake and antibiotics use history were solely based on maternal recall and may may subjected to recall bias. Finally, the relatively small sample size of the study may limit generalisability of the study findings beyond the study population.

## 5 Conclusion

In summary, this study provides a comprehensive baseline profile of mothers and newborn infants enrolled in the CHAMP cohort. The findings reveal high socioeconomic vulnerability, suboptimal antenatal and postnatal healthcare coverage, poor dietary diversity and an overall high burden of infant morbidity and early growth faltering, particularly among female infants. Suboptimal breastfeeding practices and widespread antibiotic exposure further underscore vulnerabilities during the critical early-life period. Together, these findings highlight complex interaction between maternal, infant and environmental characteristics with significant implication for the gut microbiome and infant growth and development. The CHAMP cohort study provides local evidence to inform cost effective and culturally relevant nutrition intervention strategies to improve nutritional status of the mother and infants from resource limited, high burden communities of Pakistan and neighboring countries such as Afghanistan with similar culture and dietary habits.

## Data Availability

The raw data supporting the conclusions of this article will be made available by the authors, without undue reservation.
